# Rethinking medical oxygen investments in primary health care in Nigeria

**DOI:** 10.3389/fpubh.2026.1880781

**Published:** 2026-07-20

**Authors:** Ayobami A. Bakare, Gbemisola T. Olubayo, Abiodun Sogbesan

**Affiliations:** 1 Department of Community Medicine, University College Hospital, Ibadan, Nigeria; 2 Department of Global Public Health, Karolinska Institutet, Stockholm, Sweden; 3 Oxygen for Life Initiative, Ibadan, Nigeria

**Keywords:** essential health service, health system strengthening, healthcare management, healthcare service optimization, Nigeria, primary health care (PHC)

## Introduction

Medical oxygen is widely recognized as a life-saving therapy and an essential medicine with no substitute (1). In the wake of the COVID-19 pandemic, global attention has shifted toward expanding oxygen access (2, 3), with increased investment and advocacy pushing for its inclusion even at the primary health care (PHC) level. In Nigeria, this enthusiasm requires a cautious and context-specific approach to enhance efficient and effective use. A narrative from a doctor in a Nigerian PHC reveals the high-stakes reality:

“*I saw a child with dangerously low oxygen saturation (SpO*_2_ *84%, pulse rate: 195 bpm, temperature: 38.9%, malaria rapid test: positive) who needed oxygen urgently. The concentrator had not been used for many months, so I had to retrieve it from the carton and wheeled it to the power socket in the ward and plugged it. There was no nasal prong, so we improvised using a fluid-giving set. Though other staff were hesitant to commence oxygen therapy, I pushed forward. However, as the ward maid was getting the bed ready, and the child placed in supine position, I noticed the child was very weak. With the nearest tertiary hospital just 10 mins away, I worried: was giving oxygen here the right decision? What if the child deteriorated or died here?*” *After administering pre-referral treatment, we had no choice but to refer the child, and the team was happy with the decision because a potential crisis was averted*.

PHC is the backbone of the Nigerian health system (4). It is focused on the provision of basic and essential health services. In Nigeria, PHCs are often the first—and sometimes only—contact point for many communities (5). However, the system remains underfunded, fragmented, and limited in scope mainly to preventive services and treatment of minor ailments (4). Oxygen therapy, though essential, is a complex intervention that requires reliable and functional equipment, trained and motivated staff, diagnostics like pulse oximetry, maintenance support, and an effective supply chain; many of which are lacking in Nigerian PHC facilities (6, 7). WHO's inclusion of oxygen on the Essential Medicines List underscores its global importance (8), and has amplified the call for its delivery at PHC, which is meant to provide essential health services. However, translating this into local action in primary health facilities demands context-specific strategies due to the following factors.

A closer look at oxygen services at PHC in Nigeria shows that PHC facilities are not ready for medical oxygen services. Evidence shows that pulse oximeter availability is minimal at PHC facilities, and even when oxygen equipment like cylinders or concentrators may be available, they are not frequently used (9). Studies across 146 primary health facilities in Rivers, Lagos, Kaduna, Imo, and Kano (10), and another study in primary health facilities in Abuja (11), have reported low pulse oximeter availability and oxygen use in primary health facilities. A study in Lagos shows that oxygen use and pulse oximetry at PHC are limited even with interventions (12, 13). This underscores that improving oxygen access at PHC does not automatically translate to increased oxygen use. This dichotomy between programmers or policy makers and primary health care workers needs to be resolved to ensure efficient investment in the oxygen system. We explain the reasons for poor oxygen use at primary health care and why investment in oxygen service at PHCs requires more than just increasing oxygen supply.

## Barriers to effective oxygen use at Nigerian PHCs

First, PHC in Nigeria focuses mainly on preventive care and treatment of minor ailments, provision of maternal health services like antenatal care services, family planning and management of uncomplicated deliveries. Critically ill patients—including children with hypoxemia—are expected to receive pre-referral treatment and be transferred to higher-level facilities (14). Managing such cases at PHC without adequate support risks tertiary delays and preventable deaths. Hypoxemia is a danger sign, and unlike fever or cough, it is not easily detected without pulse oximetry (15–17). Thus, the priority for Nigeria should be achieving universal pulse oximetry screening—not necessarily scale up of oxygen administration at PHC.

Second, many PHC workers are reluctant to initiate oxygen therapy. The absence of medical doctors, fear of complications, and lack of clear guidelines all contribute to oxygen hesitancy. Even when equipment and doctors are available, healthcare workers often feel unprepared or unsupported to act. This feeling is usually invisible to policy makers and program implementers, who are distal to the realities of daily clinical decision making in health facilities. In the narrative above, the doctor worried not just about the child's outcome, but about external blame if things went wrong. Understanding and incorporating healthcare provider perceptions is crucial to developing context-specific and acceptable policies that promote the adoption and integration pulse oximetry and oxygen therapy in routine primary care delivery in Nigeria.

Third, many PHCs lack 24-h coverage. Others face power instability, poor infrastructure, and security challenges. Oxygen concentrators depend on reliable electricity, and oxygen cylinders require refill systems and transport logistics. Moreover, technical personnel such as biomedical engineers are virtually absent at the PHC level. Donating oxygen equipment alone cannot overcome these constraints; in fact, past efforts have witnessed underutilization (13). A multi-sectoral approach involving power, security, health, and finance is required to strengthen and optimize the PHC system for adequate provision of medical oxygen services.

## Strategic recommendations for improved oxygen services at Nigerian PHC facilities

Besides, addressing health care workers' concerns and confidence is critical, and there is a need for clear guidelines and supporting frameworks to guide the administration of oxygen therapy at the PHCs. Existing national guidelines do not fit the PHC settings in Nigeria. Healthcare workers need to feel secure and morally justified that they are doing the right thing. Achieving this requires not just healthcare workers' training but also strategic engagement with professional bodies and training institutions to shift norms about the perception of primary healthcare among healthcare workers. We need more research work to understand the oxygen use case that fits Nigerian primary health facilities better and in what context. The use case may explore ambulatory oxygen therapy during referral or limited on-site oxygen therapy.

Therefore, to improve medical oxygen services at primary health facilities, we propose eight priority actions grouped under three broad areas: health system readiness, policy and clinical governance, and research and learning (Table 1). These pillars are drawn from contextual knowledge of PHC service delivery in Nigeria (12, 13, 18) and findings from previous studies within and outside Nigeria (18–21). The pillars reflect the systemic reforms required to ensure oxygen availability at the PHC level translates into improved patient outcomes.

**Table 1:** Strategic recommendations to improve medical oxygen services in Nigerian primary health care facilities.

Strategic pillar	Priority actions
Health System Readiness	•Expand pulse oximetry coverage across PHCs to enable early detection of hypoxemia, in line with national policy. •Strengthen PHC infrastructure and workforce capacity, including reliable power, laboratories, and essential medicines. •Enhance referral systems and functional linkages with secondary and tertiary health facilities. •Build sustainable oxygen systems at the PHC level, including equipment maintenance, power solutions, and oxygen supply chains.
Policy and Clinical Governance	•Develop clear, PHC-specific clinical guidelines on oxygen use, safety, and referral protocols. •Engage healthcare professional bodies and training institutions to integrate oxygen therapy into PHC norms and training curricula.
Research and Learning	•Pilot innovative and context-appropriate oxygen use models, such as ambulatory oxygen therapy and time-limited oxygen provision at PHC facilities. •Conduct implementation research to identify effective strategies for improving healthcare workers' confidence, motivation, and clinical decision-making in oxygen therapy.

In conclusion, expanding oxygen access in PHCs is a worthy goal, but it must be embedded within broader, system-wide reform. While Nigeria has policies and frameworks in place (6, 22), the reality on the ground is often marked by weak infrastructure, limited workforce capacity, ineffective referral linkages, and a lack of PHC-specific clinical guidelines on oxygen therapy. Critically, many healthcare workers lack the motivation, confidence, and support to initiate and manage oxygen therapy—even when equipment is available.

Nigeria must move beyond vertical, equipment-focused oxygen programs to a comprehensive, system-strengthening approach—one that ensures oxygen delivery at the PHC level is not just available, but sustainable, efficient, and lifesaving.
